# Pulmonary nodule detection on lung parenchyma images using hyber-deep algorithm

**DOI:** 10.1016/j.heliyon.2023.e17599

**Published:** 2023-06-24

**Authors:** Da Fang, Hao Jiang, Wenyang Chen, Zhibao Qin, Junsheng Shi, Jun Zhang

**Affiliations:** a School of Physics and Electronic Information, Yunnan Normal University, Kunming 650500, China; b Yunnan Key Laboratory of Optoelectronic Information Technology, Kunming 650500, China

**Keywords:** Lung nodule detection, Information security, Transfer learning, Internet of things

## Abstract

The incidence of lung cancer has seen a significant increase in recent times, leading to a rise in fatalities. The detection of pulmonary nodules from CT images has emerged as an effective method to aid in the diagnosis of lung cancer. Ensuring information security holds utmost significance in the detection of nodules, with particular attention given to safeguarding patient privacy within the context of the Internet of Things (IoT). In this regard, migration learning emerges as a potent technique for preserving the confidentiality of patient data. Firstly, we applied several data-preprocessing steps such as lung segmentation based on K-Means, denoising methods, and lung parenchyma extraction through a dedicated medical IoT network. We used the Microsoft Common Object in Context (MS-COCO) dataset to pre-train the detection framework and fine-tuned it with the Lung Nodule Analysis 16 (LUNA16) dataset to adapt to nodule detection tasks. To evaluate the effectiveness of our proposed pipeline, we conducted extensive experiments that included subjective evaluation of detection results and quantitative data analysis. The results of these experiments demonstrated the efficacy of our approach in accurately detecting pulmonary nodules. Our study provides a promising framework for trustworthy pulmonary nodule detection on lung parenchyma images using a secured hyper-deep algorithm, which has the potential to improve lung cancer diagnosis and reduce fatalities associated with it.

## 1 Introduction

According to authoritative statistical figures from the World Health Organization (WHO) and major cancer research centers, lung cancer is the leading cause of cancer-related deaths worldwide [1]. In China, lung cancer ranked first with approximately 870,000 confirmed cases and nearly 760,000 fatalities, underscoring its importance in medical security [2]. Thus, lung cancer constitutes a crucial focal point that demands attention within the purview of medical safety.

Lung nodule detection is an essential prerequisite for diagnosing lung cancer [3]. The measurement and characterization of pulmonary nodules are performed using continuous radiological images. However, the annotation process can be time-consuming and prone to error, which underscores the need for accelerating the reading speed of CT scans [1,4]. Computer-aided diagnosis (CAD) technology can address this issue by facilitating rapid and accurate labeling of lung nodules, thereby helping radiologists diagnose the disease while reducing their burden [1]. Timely identification of individuals at risk is of paramount importance [5], as it enables prompt intervention and management of the condition.

The escalating concerns surrounding IoT security have brought information security to the forefront, particularly when addressing the amalgamation of IoT security, privacy, and healthcare security [6]. Within the realm of diagnosis, the security of patient information assumes paramount importance. Hence, it is imperative to safeguard medical CT equipment, image transmission data, and systems against potential threats and breaches, ensuring the privacy of medical information, and guaranteeing the accuracy and confidentiality of lung nodule detection outcomes warrant thoughtful consideration.

To achieve a computer-aided diagnosis system that encompasses secure data transmission and storage, the transmission and storage of CT images in the IoT system demand careful attention [7]. Ensuring the security of the data transmission channel becomes critical to prevent unauthorized tampering, data theft, or leakage during transmission or storage, especially when images are transmitted over networks or stored in cloud platforms.

Deep learning has emerged as a highly effective tool for analyzing various medical images [8]. As such, there is a growing interest in exploring different neural networks for computer-aided diagnosis of lung diseases. Transfer learning, a method that leverages pre-existing solution models for related but distinct problems, gains prominence in this context [9]. The process of training the model through transfer learning can be described as follows: Initially, the pre-trained model undergoes fine-tuning using patient data. Subsequently, the fine-tuned model is employed as the output, ensuring the preservation of patient privacy by avoiding direct involvement of patient-specific data. Moreover, in the context of a computer-aided diagnosis system, hospitals have the option to input data into the pre-trained model for continued training. This approach not only safeguards the privacy of remote patient data but also ensures the secure handling of patient information, preventing potential data leakage.

Notably, research has shown that the use of low-dose computed tomography (LDCT) can reduce the specific mortality rate of lung cancer cells by 20% compared to X-rays [10]. Furthermore, the United States Preventive Services Task Force (USPSTF) recommends annual surveillance of people with lung disease using LDCT [3], highlighting its potential for lung cancer detection [11]. Building on prior work, this paper leverages LDCT images to accurately localize patients with pulmonary nodules.

While 3D-based neural networks have achieved satisfactory performance in detecting pulmonary nodules in CT images, they can be problematic due to the variable slice thickness of CT scans, which can cause information leakage in the reconstructed 3D images. Additionally, the use of 3D structures requires more time and resources for training, making them less practical for widespread use. In light of these issues, the authors of this paper have proposed a novel framework for automatic lung nodule detection using a 2D convolutional neural network (CNN) to facilitate the process of detecting these abnormalities in CT images. By utilizing a 2D CNN, the authors aim to overcome the limitations of 3D-based approaches and create a more efficient and effective method for detecting lung nodules.

Radiomics has emerged as a powerful tool for detecting tumor phenotypes by utilizing quantitative image features. This technique enables more refined patient classification, ultimately facilitating tailored cancer treatments. Specifically, radiomics involves segmenting the region of interest (ROI) in radiological images [12] and deriving relevant image features through automated analysis using machine learning methods [13]. The present article describes the scientific methodology of radiomics, whereby radiologists first outline the handcrafted feature using Slicer [14], before training image data using the combined advantages of convolutional neural networks [5].

## 2 Related work

The present study sheds light on prior investigations that employed diverse techniques to accurately categorize lung nodules. Firstly, a CAD method was developed, which stratified the nodules into three main groups, namely metastatic lung cancer, primary lung cancer, and benign nodules [15]. Secondly, neural networks, specifically convolutional neural networks (CNN), have been widely adopted for the purpose of detecting lung nodules. Notably, the literature reports the successful classification of 311 individuals suffering from early-stage non-small cell lung cancer (NSCLC) using CNNs for histological subtyping [14]. Furthermore, research efforts have focused on enhancing the classification accuracy of pulmonary nodules as benign or malignant, utilizing an improved residual convolutional neural network (IRCNN) [16].

Prior investigations have explored the utilization of modified multi-discriminator Generative Adversarial Networks (GAN) in generating models for recognition, which employ unsupervised learning. The performance of these models was found to be superior to that of other supervised mechanisms [8]. Moreover, the literature describes an automated approach for the detection of pulmonary nodules using 3D U-Net convolutional neural networks (CNNs) based on transfer learning, which incorporates multiscale characteristics [13]. Besides, it has been established that a single model or architecture may not always yield the optimal solution. In this regard, the literature suggests that a prospective strategy for training convolutional neural networks (CNNs) involves fine-tuning [9].

Besides, the present study extends the utility of the MM-Detection library for the purposes of model fine-tuning. This library is a comprehensive toolkit for object detection, comprising both training and imputation code for weight initialization of network models, as well as an assortment of detection methods and related components. The MM-Detection library has gained significant traction, as it offers a unified platform that encompasses prevailing detection methods [17].

In the domain of detecting and segmenting lung nodules, various architectures and models have been implemented. For instance, Markov random field (MRF) models have been applied for the segmentation of lung CT images to identify ground glass turbidity [18]. In addition, two customized mixed link network (CMixNet) frameworks have been proposed in the literature for lung nodule detection and sorting [11]. Moreover, a fully convolutional one-stage object detector (FCOS) has been proposed in the literature [19]. This method performs object detection by pixel forecasting, eliminating the need for pre-defined fixed anchor boxes and, in turn, avoiding the associated computational complexity. Compared to relying on pre-defined anchor boxes, FCOS presents a simpler and more agile detection framework, achieving superior detection accuracy. Another multi-phase approach for object detection, the Cascade *R*–CNN, has been proposed in the literature [20]. This framework is trained by inspecting detected objects with augmenting intersection over union (IOU) thresholds and has been previously shown to outperform single-model target detection using COCO. These two architectures are compared in terms of performance superiority in the present study.

The literature suggests that the COCO dataset is an essential resource for target recognition methods, offering a comprehensive collection of images from diverse environments. It encompasses 91 object categories with over 2.5 million annotated instances in 328,000 images [21], and is commonly employed for object detection tasks. Therefore, this study employs COCO as a pre-training dataset for lung nodule detection. The LUNA16 dataset, which includes 888 CT scans, is publicly available and widely used for the evaluation of nodule detection algorithms [22]. The authors employ COCO for pre-training and fine-tune it using the LUNA16 dataset as a calibration set after lung parenchymal segmentation. Finally, the LUNA16 and multiple primary lung cancer (MPLC) datasets serve as the test set for the evaluation of the proposed method.

For lung nodule detection, this paper achieves the neural network trained by LUNA16 achieved an average mAP50 of 60.11%, Recall of 75.63%, and Speed of 9.5000 task/s. The neural network fine-tuned by LUNA16 reached an average mAP50 of 63.71%, Recall of 74.34%, and Speed of 8.9000 task/s. Upon analyzing the two datasets, it is evident that the utilization of fine-tuning techniques has resulted in a discernible improvement in the performance of the pre-training model.

## 3 Methodology

The conventional process for training neural networks usually necessitates a vast collection of annotated datasets. Nonetheless, for research domains that are not mainstream, creating an annotated dataset is a costly and laborious process. Therefore, Transfer Learning [9] and Few-Shot learning have been suggested as alternative techniques to cope with this challenge. These methods enable the transfer of knowledge from a pre-trained model to a new task or the learning of a new task with a limited number of annotated samples, respectively.

In the Stage 1: Lung Segmentation section, data preprocessing is performed to obtain parenchymal lung images after erosion and expansion of the input images.

In the Stage 2: Nodule Detection section: the LUNA16 dataset is used to fine-tune the network architecture and the Backbone is replaced with Dark-Net53 to finally achieve the detection results.

The obtained detection results are subjected to both qualitative evaluation and quantitative calculation to comprehensively assess their efficacy and performance, respectively.

**Fig. 1:**
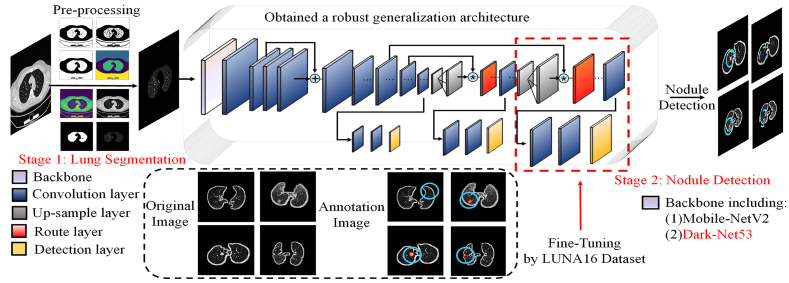
The overall nodule detection procedure of the proposed pipeline [[17], [23], [24]].

Henceforth, the task of detecting nodules exhibits a positive correlation between object detection and instance segmentation. Moreover, the utilization of deep learning and artificial detection methods for nodule detection presents challenges of data sparsity and computational complexity, leading to a time-consuming process. To address these issues and enable automated and efficient nodule screening, we present a novel detection methodology illustrated in Fig. 1. Therefore, the pseudo code of algorithm 1 had shown the detection procedure of nodule detection. S represents the grid size, B denotes the box, the complete loss function was composed with the loss of bbox, the loss of the confidence coefficient, and the loss of the category.

(1) $$Loss_{v3}=\lambda_{cord}Err_{CCE}+\lambda_{cord}Err_{WHCE}+Err_{EC}+Err_{ECla}$$

In equation (1), the $Err_{CCE}$ means the center coordinate error; $Err_{WHCE}$ represents the width and height coordinate error, $Err_{EC}$ denotes the error of confidence, and tells the error of classification. To denoise the source image, lung segmentation is a valuable technique. Besides, to finish the nodule detection tasks with Few-shot datasets. Besides, the neural networks are pre-trained by the MS-COCO dataset [21], which can prove an essential detection ability for the trained model. Thus, to domain the specific detection targets, the proposed pipeline applied the LUNA16 dataset [22] to fine-tune the pre-trained models. In section 4, the comparison of the proposed methods was shown by quantitative results and qualitative analysis.

**Table:** 

Algorithm1: Nodule Detection by YOLOv3
**1: Input**←Output←mask+middle_image 2: IoU=|A∩B|/|A∪B| 3: $GIoU=IoU-\left|A_{c}-U\right|/\left|A_{c}\right|$ 4: $\left[x,y,w,h\right]\leftarrow \left[{\left(x_{i}-{\hat{x}}_{i}\right)}^{2}+{\left(y_{i}-{\hat{y}}_{i}\right)}^{2}+{\left(w_{i}-{\hat{w}}_{i}\right)}^{2}+{\left(h_{i}-{\hat{h}}_{i}\right)}^{2}\right]$ 5: $\sum_{yolov3}\leftarrow \sum_{i=0}^{S^{2}}\sum_{j=0}^{B}1_{i,j}^{object}$ **6: if class** = **1://Nodule detection: class** = **1** **7:**lclass=0 **8: else:** **9:**$lclass=\lambda_{class}\sum_{yolov3}\sum_{c\in classes}p_{i}\left(c\right)\log\left(\hat{p_{i}}\left(c\right)\right)$ **10: end if** 11: $lbox=\lambda_{coord}\sum_{yolov3}\left(2-w_{i}h_{i}\right)\left[x,y,w,h\right]$ 12: $lobj=\lambda_{noobj}{\sum_{yolov3}\left(c_{i}-{\hat{c}}_{i}\right)}^{2}+\lambda_{noobj}{\sum_{yolov3}^{obj}\left(c_{i}-{\hat{c}}_{i}\right)}^{2}$ 13: $loss_{yolov3}=lbox+lobj+lcls$

$$\begin{array}{l} bbox_{x}=\sigma \left(t_{x}\right)+c_{x}\\ bbox_{y}=\sigma \left(t_{y}\right)+c_{y}\\ bbox_{w}=p_{w}e^{t_{w}}\\ bbox_{h}=p_{h}e^{t_{h}} \end{array}$$

We will divide each image into N*N grids, $c_{x}c_{y}$ refers to the number of grids where the upper left corner of the point is from the upper leftmost corner; $p_{w}p_{h}$ refers to the edge length of the a priori frame; $t_{x}t_{y}$ refers to the offset of the target centroid relative to the upper left corner of the grid where the point is located; $t_{w}t_{h}$ refers to the width and height of the predicted frame; σ refers to the activation function, using the sigmoid function; $bbox_{x}bbox_{y}$ refers to the coordinates of the bounding box; $bbox_{w}bbox_{h}$ refers to the width and height of the bounding box; finally, this results in the position of the bounding box [23].

**Table:** 

Algorithm2: Data Pre-processing
**1: Input**$\leftarrow Source\;CT\;images:X=\left\{x_{1},...,x_{n}\right\}$ **2: for**i←1 to n**do:** 3: $K-Means:E=\sum_{i=1}^{k}\sum_{x\in C_{i}}\|x-u_{i}|{|}_{2}^{2}$, $u_{i}=\frac{1}{\left|C_{i}\right|}\sum_{x\in C_{i}}x$; 4: src1←K−Means(Input)// K−Meansforremoveprospect 5: src2←erode(src1,dst1,elem) **6: While**erosion==0:// elem←EroRec,EroCro,EroEll **7:**EroRec(src1) **8: if**erosion==1: **9:**EroCro(src1) **10: if**erosion==2: 11: EroEll(src1) **12: end if** **13:**src2←dsr1=erode(src1) 14: e**nd while** 15: Middle_image←dilation(src2,dst2,elem) **16: While**dilattion==0:// elem←DilRec,DilCro,DilEll **17:**DilRec(src2) **18: if**dilattion==1: **19:**DilCro(src2) **20: if**dilattion==2: 21: DilEll(src2) **22: end if** **23:**middle_image←dsr2=dilate(src2) 24: **end while** 25: mask←BinnaryMask(Input) **26: end for** 27: Output←mask+middle_image

**Fig. 2:**
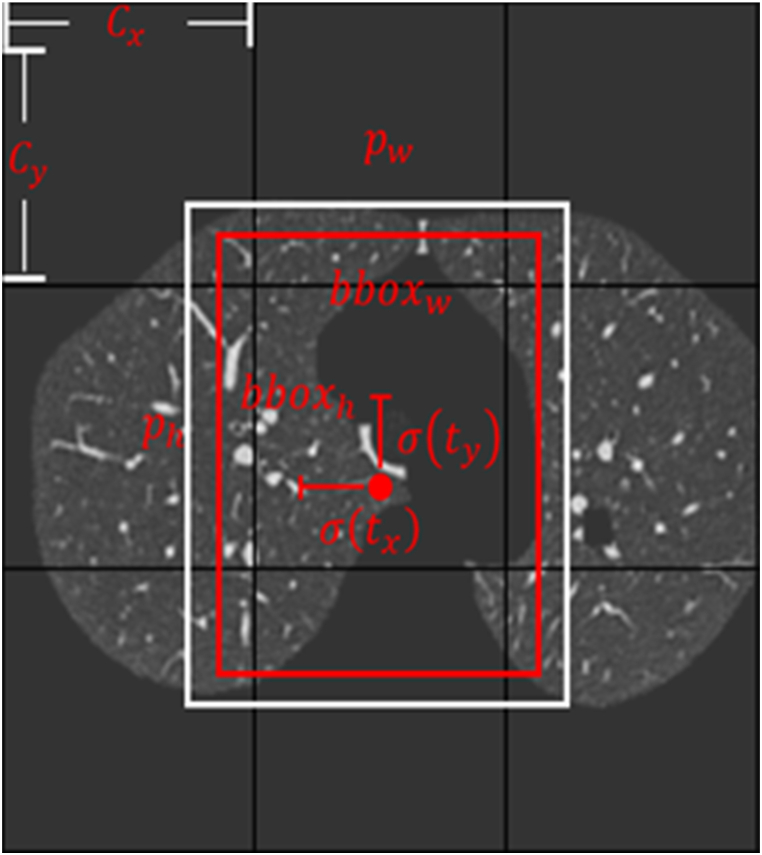
The boundary-box (bbox) was used to detect pulmonary nodules.

Data pre-processing serves as an excellent approach to enhance the behavior of the training procedure. Algorithm 2 in this paper is called: lung parenchyma extraction. It includes serval processing steps: 1) The prospects are separated by the K-Means technique; 2) The erosion and dilation are applied to remove specific areas of particles and pulmonary vessels; 3) The extraction of lung masks. After undergoing pre-processing procedures, the neural network effectively removes any background noise, reduces the detection area, optimizes the data, and minimizes resource consumption for nodule detection. These techniques aid in enhancing the neural network's performance in accurately identifying nodules. The above pre-processing detail is shown in Fig. 2. The pre-processing equations are as follows:

(2) $$\mathrm{Min}\_E=\sum_{i=1}^{k}\sum_{X\in C_{i}}\|X-U_{i}|{|}_{2}^{2},U_{i}=\frac{1}{\left|C_{i}\right|}\sum_{X\in C_{i}}X$$

(3) $$MiddleImage=f_{Dilation}f_{Erosion}\left(K-Means\left(CT\right)\right)$$

(4) Output=Fusion(CT+lungmask(Middle_Image))

The algorithm of K-Means is to minimize the distances of samples from the cluster center $U_{i}$, as shown in Equation (2). Besides, the similarity within the cluster is a negative correlation E. Thus, applying the dilation and erosion method on the processed images removed prospects by the K-Means algorithm, as shown in Equation (3). In this way, the particles and vessels of images can be removed. Finally, extracting lung mask from the middle image to fusion with the source images, as shown in Equation (4). The lung segmentation tasks can be finished to denoise. CT means the source images of lungs. Meanwhile, the pseudo code of data-preprocessing was introduced in the above, which could detailed describe the pre-processing procedure.

**Fig. 3:**
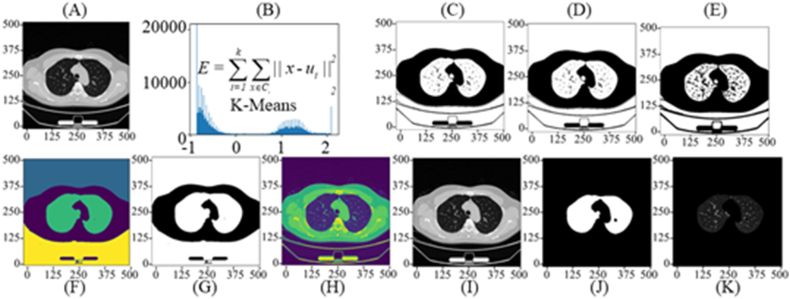
The data pre-processing—lung segmentation for nodule detection.

Pre-Processing Schedule: Fig. 3 (A-B) Normalization; Fig. 3 (C) Back ground-foreground separation; Fig. 3 (D-G) Erosion and dilation: 1) Erosion is to wipe off particle; 2) Dilation is to engulf vessel; 3) Remove black noise especially blacklung areas caused by opaque rays. Fig. 3 (H–K): Extracting the mask of lungs.

The study employed MM-Detection as the platform for training, which utilized the Tesla-V100 GPU, while MATLAB 2022B was used for lung extraction. The pre-processing and training steps for additional data were conducted on an NVIDIA 3060 GPU and an Intel i7 10400f.

The pulmonary nodule detection dataset employed in this research consists of the LUNA16 and a private dataset. The nodule dataset was formatted using VOC2007 to suit the MM-Detection platform's application.

## 4 Results

**Fig. 4:**
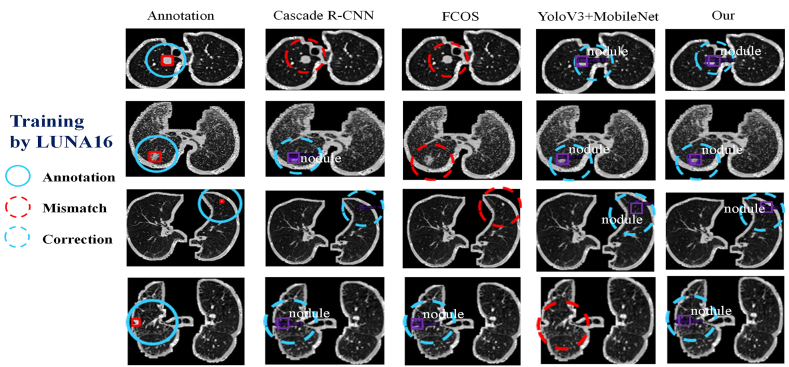
The Detection Result is based on Four Architectures (Trained on LUNA16 Dataset).

To objectively evaluate the effectiveness of the proposed pipeline in comparison to other widely used object detection frameworks, this study selected the FCOS [19], Cascade *R*–CNN [20], and YOLOv3 (backbone is Mobile-Net) [[17], [23], [24]] as the control groups. Both qualitative and quantitative assessments were conducted on the experimental results, which are presented in Fig. 4. The LUNA16 dataset was used to train all four detection methods. The findings revealed that the proposed method can accurately locate the nodule coordinates. However, due to the limited availability of pulmonary nodule datasets, the trained model still faces certain challenges, including high false positives, missed nodules, and incorrectly predicted nodule locations when compared with annotations.

**Fig. 5:**
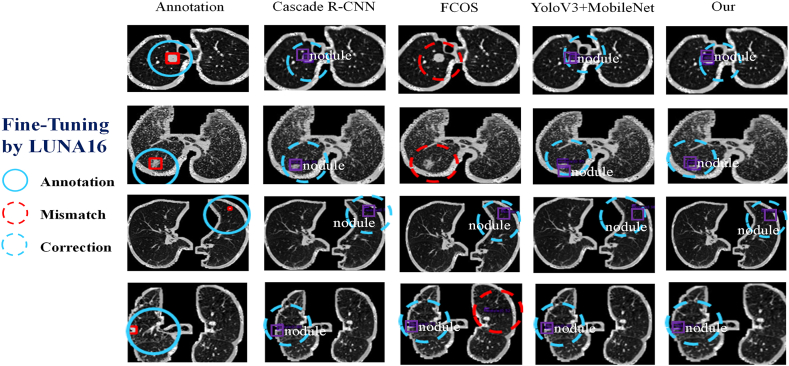
The Detection Result is based on Four Architectures (Fine-Tuned on LUNA16 Dataset).

The objective of this study is to enhance the detection performance of training programs. To this end, a Transfer-learning procedure is employed on four neural networks. The pre-trained architecture is obtained using the MS-COCO dataset, which serves as evidence for the fundamental detection ability of object detection. Subsequently, the present study endeavors to enhance the performance of pre-trained models through the fine-tuning technique. The pre-trained model is fine-tuned using the LUNA16 dataset, taking into account the peculiarities of lung nodules. The detection outcomes are depicted in Fig. 5.

**Fig. 6:**
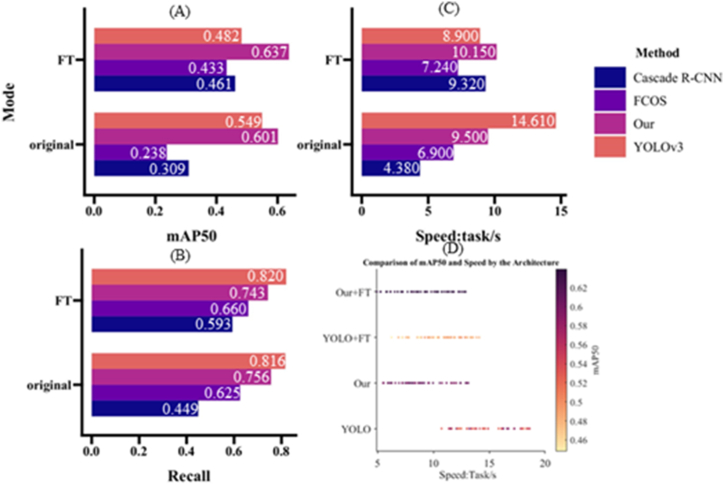
The quantitative evaluation results of accuracy of the neural networks.

**Fig. 7:**
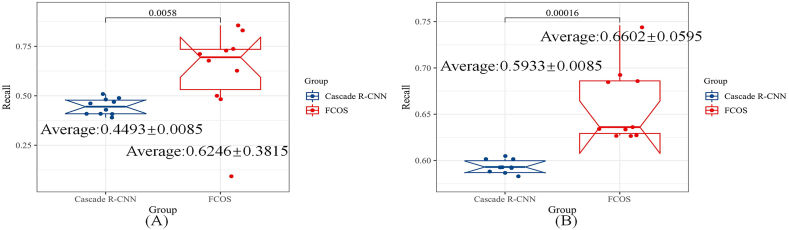
The comparison of recall for the FCOS and the cascade R–CNN

**Fig. 8:**
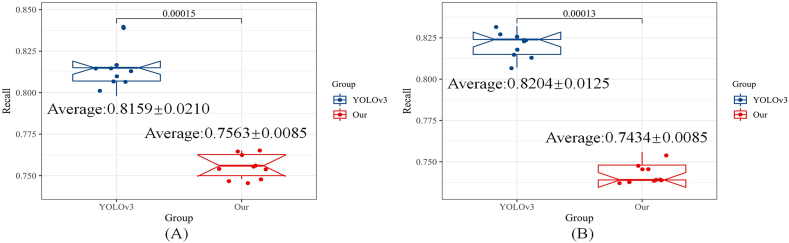
The comparison of recall for the YOLOv3 and the proposed pipeline.

Despite the presence of mismatch situations, the fine-tuning technique enables the detection of a considerable number of nodules in accurate coordinates. This observation is reinforced by the quantitative analysis results, which are presented in Fig. 6, Fig. 7, Fig. 8.

The paper presents an evaluation of three metrics, namely mAP50, as shown in Equation (7), Detection Speed, and Recall, as shown in Equation (5), in the context of nodule detection using various models. Specifically, the authors compare the performance of the Fine-Tuned YOLOv3 (backbone is DarkNet53) in eight different training conditions against other models, including Cascade *R*–CNN, YOLOv3 (backbone is Mobile-Net), and YOLOv3 (backbone is DarkNet53) except FCOS. The evaluation is carried out on two groups, the original (only training by LUNA16) and the Fine-tuned (by LUNA16) groups, as depicted in Fig. 6(A)–(C), where the Y-axis represents the group composition.

(5) $$Recall=\frac{TruePositive}{TruePositive+FalseNegative}=\frac{TruePositive}{GroundTruth}$$

(6) $$IOU=\frac{Area\_of\_Overlap}{Area\_of\_Union}$$

(7) $$mAP=\frac{1}{N}\sum_{i=1}^{N}AP_{i}$$

The results indicate that the Fine-Tuned YOLOv3 achieves the best mAP50 value in all eight training conditions, and the fine-tuning techniques improve mAP50 values for all models except FCOS. Additionally, the authors compare the performance of four different methods in terms of mAP50 and detection speed, as shown in Fig. 6 (D), where the Fine-tuned YOLOv3 (backbone is DarkNet53) outperforms the other methods in both mAP50 and detection speed. Overall, Fig. 6 provides objective results demonstrating that the proposed pipeline can achieve faster and more precise nodule detection tasks, albeit with some time-consuming issues. Specifically, the proposed method achieved the best mAP50 value for both groups and second place in Recall value for both groups, whereas it only achieved third place among the four methods in terms of time consumption, as reflected in Fig. 6 (C).

The IOU denotes the discussion of the Intersection and union of prediction region and label region, as shown in Equation (6). Besides, as calculating the IOU value in the YOLOv3, it only pays attention to the width and height. Therefore, $mAP_{50}$ equal to the value of IOU is 50%. The Recall value means the ability to discover, but Precision reflects the capacity to locate nodules. The complete calculating procedure was introduced in the pseudo code of algorithm 1. However, the mAP50 is the comprehensive metric that combines the peculiarity of Precision and Recall. Therefore, this paper is designed to illustrate the efficiency of transfer learning techniques. The comparison of each method based on the Recall value is shown in Fig. 7, Fig. 8.

In Fig. 7, Fig. 8 depicts the original architecture trained on the LUNA16 dataset, while Fig. 7, Fig. 8 represents the fine-tuned models. The experimental results in these figures demonstrate that Transfer-Learning techniques can significantly enhance the detection ability of the four methods. However, the proposed pipeline yields a slightly lower Recall value compared to the original YOLOv3, albeit the difference between the two methods is negligible.

## 5 Discussion

To provide evidence of the proposed method's efficacy in the nodule detection task, this research paper presents the findings in Table 1 and Table 2. These tables primarily focus on quantitative metrics, namely mAP50, recall, and detection speed, to comprehensively assess the performance. The aggregated results from both tables consistently demonstrate the superiority of our model, both before and after fine-tuning.

**Table 1:** The quantitative evaluation results of neural networks, trained by LUNA16.

Metric/Method	Cascade *R*–CNN	FCOS	YoLov3+MobileNet	Our
mAP50	0.3090 ± 0.0043	0.2377 ± 0.2114	0.5489 ± 0.0379	**0.6011** ± **0.0101**
Recall	0.4493 ± 0.0085	0.6246 ± 0.3815	**0.8159** ± **0.0210**	0.7563 ± 0.0085
Speed: task/s	**4.3800** ± **0.0500**	6.9000 ± 0.0500	14.610 ± 0.0500	9.5000 ± 0.0500

**Table 2:** The Quantitative Evaluation Results of Neural Networks, which Fine-Tuned by LUNA16.

Metric/Method	Cascade *R*–CNN	FCOS	YoLov3+MobileNet	Our
mAP50	0.4609 ± 0.0024	0.4334 ± 0.0671	0.4820 ± 0.0258	**0.6371** ± **0.0018**
Recall	0.5933 ± 0.0085	0.6602 ± 0.0595	**0.8204** ± **0.0125**	0.7434 ± 0.0085
Speed: task/s	9.3200 ± 0.0500	**7.2400** ± **0.0500**	10.150 ± 0.0500	8.9000 ± 0.0500

**Fig. 9:**
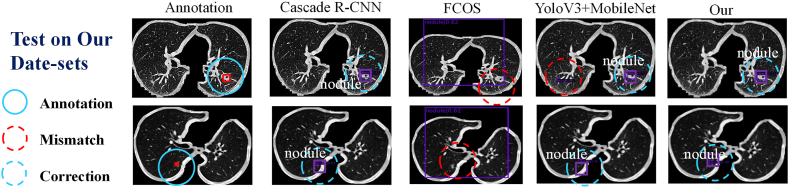
The Detection Result based on Neural Network on Our MPLC Dataset.

Additionally, qualitative results are showcased through the nodule detection outcomes depicted in Fig. 9. The figure presents different categories: Annotation, which aids in the observation; Mismatch, indicating instances where the model failed to detect nodules; and Correction, denoting successful detections. The graphical results clearly indicate the superiority of our proposed model. It is important to note that both the qualitative and quantitative evaluations are based on the MPLC dataset. The MPLC dataset focuses on two-dimensional radiological features and specifically investigates nodal detection tasks.

Notably, MPLC refers to the presence of two or more primary malignant masses within the same patient's lungs. The selection of the MPLC dataset is significant as it is a valuable resource, considering that only a few lung cancer patients exhibit multiple primaries. The dataset originates from Yunnan Province, China, enhancing its practical significance in advancing medical research. However, a limitation of this evaluation is the use of a private dataset instead of a public one. Therefore, it is recommended to include a public CT dataset of lung cancer as an additional dataset for subsequent evaluations.

It is imperative to emphasize the importance of ensuring data processing and analysis safety during the detection of lung nodules. The handling of private patient data involves the processing and analysis of CT images, as well as the consideration of patient identities and case details, necessitating the implementation of privacy protection measures. Encryption technology plays a crucial role in maintaining image confidentiality and anonymizing or desensitizing personally identifiable patient information. By employing such measures, unauthorized data access and disclosure can be effectively prevented [6], thereby ensuring the safety and security of these processes and analyses.

In certain scenarios where remote access and collaboration are required, physicians may need to access CT images and relevant data or collaborate with other medical specialists. Migration learning-trained models offer a viable solution to address this requirement. By securely sharing the trained models subsequent to guaranteeing the platform's security [25], including measures to prevent unauthorized access, data leakage, and malicious data tampering [26], the information challenge associated with IoT security and privacy can be practically addressed.

## 6 Conclusion

The present paper presents a novel pipeline for precise assessment of nodular detection tasks in a two-dimensional context, thereby enabling the convergence of intelligent healthcare and the Internet of Things. The pipeline utilizes Transfer-learning and lung segmentation pre-processing steps to fine-tune the fundamental YOLOv3 with DarkNet-53, resulting in the best mAP50 value and second-best Recall value. Additionally, the proposed pipeline satisfies the need for detection speed. However, there are several drawbacks to the proposed pipeline that require further attention. These include the weak relevance between MS-COCO and LUNA16, the recent release of improved YOLOv3-YOLOV5, and the possibility of accidently deleting nodules during data pre-processing. Therefore, future research endeavors can concentrate on augmenting the proposed pipeline to attain superior efficacy.

In addition, some limitations of the study are as follows.

1. Although the model achieved the best mAP50 value, there is still a deficiency in the recall rate, 74.34% is still a space with a part of progress to improve the accuracy of detection.
2. The neural network in this paper is pre-trained by MS-COCO [21] dataset, which is not the most suitable pre-training dataset for medical image processing.
3. YOLOv3 may be a bit outdated for the current model, and it would be better to compare with the new model.
4. There are only three quantitative evaluation indicators, which are relatively few and can be increased some more.

Furthermore, it is crucial to address the security concerns pertaining to deep learning models, given their pivotal role in lung nodule detection. Within the IoT framework, robust measures should be implemented to prevent tampering, malicious manipulation, and safeguard the integrity of data during both model training and detection stages [27]. The overarching objective is to establish a medical diagnostic service that is characterized by heightened security, reliability, and privacy protection. By ensuring the privacy and security of CT image data, alongside fortifying the security and reliability of deep learning models, we can elevate the healthcare experience for patients, safeguard their privacy, and enhance overall healthcare safety.

## Ethics statement

The study on patients or volunteers has been approved by the ethics committee and informed consent has been obtained.

The ethics committee: Ethics Committee of the First People's Hospital of Yunnan Province.

## Author contribution statement

Da Fang: Conceived and designed the experiments; Performed the experiments; Analyzed and interpreted the data; Wrote the paper.

Hao Jiang; Wenyang Chen: Performed the experiments.

Junsheng Shi; Jun Zhang; Zhibao Qin: Contributed reagents, materials, analysis tools or data.

## Data availability statement

The authors do not have permission to share data.

## Declaration of competing interest

The authors declare that they have no known competing financial interests or personal relationships that could have appeared to influence the work reported in this paper

## References

[1] Ren H., Zhou L., Liu G., Peng X., Shi W., Xu H., Shan F., Liu L. (2020). An un-supervised semi-automated pulmonary nodule segmentation method based on enhanced region growing. Quant. Imag. Med. Surg..

[2] Xia C., Dong X., Li H., Cao M., Sun D., He S., Yang F., Yan X., Zhang S., Li N., Chen W. (2022). Cancer statistics in China and United States, 2022: profiles, trends, and determinants. Chinese Med J.

[3] Ali I., Hart G.R., Gunabushanam G., Liang Y., Muhammad W., Nartowt B., Kane M., Ma X., Deng J. (2018). Lung nodule detection via deep reinforcement learning. Front. Oncol..

[4] Xie Y., Xia Y., Zhang J., Song Y., Feng D., Fulham M.J., Cai W.T. (2019). Knowledge-based collaborative deep learning for benign-malignant lung nodule classification on chest CT. IEEE Trans. Med. Imag..

[5] Xie H., Yang D., Sun N., Chen Z., Zhang Y. (2019). Automated pulmonary nodule detection in CT images using deep convolutional neural networks. Pattern Recogn..

[6] Meng W., Li W., Wang Y., Au M. (2020). Detecting insider attacks in medical cyber-physical networks based on behavioral profiling. Future Generat. Comput. Syst..

[7] Meng W., Cai Y., Yang L.T., Chiu W. (2021). Hybrid emotion-aware monitoring system based on brainwaves for internet of medical things. IEEE Internet Things J..

[8] Kuang Y., Lan T., Peng X., Selasi G.E., Liu Q., Zhang J. (2020). Unsupervised multi-discriminator generative adversarial network for lung nodule malignancy classification. IEEE Access.

[9] Tajbakhsh N., Shin J.Y., Gurudu S.R., Hurst R.T., Kendall C.B., Gotway M.B., Liang J. (2016). Convolutional neural networks for medical image analysis: full training or fine tuning?. IEEE Trans. Med. Imag..

[10] Zhang C., Sun X., Dang K., Li K., Guo X.w., Chang J., Yu Z.q., Huang F.y., Wu Y.s., Liang Z. (2019). Toward an expert level of lung cancer detection and classification using a deep convolutional neural network. Oncol..

[11] Nasrullah N., Sang J., Alam M.S., Mateen M., Cai B., Hu H. (2019). Automated lung nodule detection and classification using deep learning combined with multiple strategies. Sensors.

[12] Fave X., Mackin D., Lee J., Yang J., Zhang L. (2016). Computational resources for radiomics. Transl. Cancer Res..

[13] Tang S., Yang M., Bai J. (2020). Detection of pulmonary nodules based on a multiscale feature 3D U-Net convolutional neural network of transfer learning. PLoS One.

[14] Chaunzwa T.L., Hosny A., Xu Y., Shafer A., Diao N., Lanuti M., Christiani D.C., Mak R.H., Aerts H.J. (2021). Deep learning classification of lung cancer histology using CT images. Sci. Rep..

[15] Nishio M., Sugiyama O., Yakami M., Ueno S., Kubo T., Kuroda T., Togashi K. (2018). Computer-aided diagnosis of lung nodule classification between benign nodule, primary lung cancer, and metastatic lung cancer at different image size using deep convolutional neural network with transfer learning. PLoS One.

[16] Afag S. (2020). Classification of lung nodules using improved residual convolutional neural network. J. Computat. Sci. Intellig. Technol..

[17] Chen K., Wang J., Pang J., Cao Y., Xiong Y., Li X., Sun S., Feng W., Liu Z., Xu J., Zhang Z., Cheng D., Zhu C., Cheng T., Zhao Q., Li B., Lu X., Zhu R., Wu Y., Dai J., Wang J., Shi J., Ouyang W., Loy C.C., Lin D. (2019).

[18] Zhu Y., Tan Y., Hua Y., Zhang G., Zhang J. (2012). Automatic segmentation of ground-glass opacities in lung CT images by using markov random field-based algorithms. J. Digit. Imag..

[19] Tian Z., Shen C., Chen H., He T. (2019). 2019 IEEE/CVF International Conference on Computer Vision (ICCV).

[20] Cai Z., Vasconcelos N., Cascade R.-C.N.N. (2018). 2018 IEEE/CVF Conference on Computer Vision and Pattern Recognition.

[21] Lin T.Y., Maire M., Belongie S.J., Hays J., Perona P., Ramanan D., Dollar P., Zitnick C.L. (2014). European Conference on Computer Vision.

[22] Setio A.A.A., Traverso A., de Bel T., Berens M.S.N., van den Bogaard C., Cerello P., Jacobs C. (2017). Validation, comparison, and combination of algorithms for automatic detection of pulmonary nodules in computed tomography images: the luna16 challenge. Med. Image Anal..

[23] Redmon J., Farhadi A. (2018).

[24] Choi J., Chun D., Kim H., Lee H.-J. (2019). 2019 IEEE/CVF International Conference on Computer Vision (ICCV).

[25] Lin G., Wen S., Han Q., Zhang J., Xiang Y. (2020). Software vulnerability detection using deep neural networks: a survey. Proc. IEEE.

[26] Wang M., Zhu T., Zhang T., Zhang J., Yu S., Zhou W. (2020). Security and privacy in 6G networks: new areas and new challenges. Digital Communic. Networks.

[27] N. Sun, M. Ding, J. Jiang, W. Xu, X. Mo, Y. Tai, J. Zhang, Cyber Threat Intelligence Mining for Proactive Cybersecurity Defense: A Survey and New Perspectives, IEEE Communications Surveys & Tutorials.

